# Exploring the *Silent* Aspect of Carbon Nanopores

**DOI:** 10.3390/nano11020407

**Published:** 2021-02-05

**Authors:** Teresa J. Bandosz

**Affiliations:** Department of Chemistry and Biochemistry, The City College of New York, 160 Convent Avenue, New York, NY 10031, USA; tbandosz@ccny.cuny.edu

**Keywords:** nanoporous carbons, ultramicropores, adsorption potential, oxygen reduction, CO_2_ reduction, sensing, surface chemistry

## Abstract

Recently, owing to the discovery of graphene, porous carbons experienced a revitalization in their explorations. However, nowadays, the focus is more on search for suitable energy advancing catalysts sensing, energy storage or thermal/light absorbing features than on separations. In many of these processes, adsorption, although not emphasized sufficiently, can be a significant step. It can just provide a surface accumulation of molecules used in other application-driving chemical or physical phenomena or can be even an additional mechanism adding to the efficiency of the overall performance. However, that aspect of confined molecules in pores and their involvement in the overall performance is often underrated. In many applications, nanopores might *silently* advance the target processes or might very directly affect or change the outcomes. Therefore, the objective of this communication is to bring awareness to the role of nanopores in carbon materials, and also in other solids, to scientists working on cutting-edge application of nonporous carbons, not necessary involving the adsorption process directly. It is not our intention to provide a clear explanation of the small pore effects, but we rather tend to indicate that such effects exist and that their full explanation is complex, as complex is the surface of nanoporous carbons.

## 1. Introduction

Carbons are one of the oldest man-made materials known to the humans. Some sources indicated that they have been used in 3750 B.C for ores smelting and constructions along the River Nile. Beside the advantage of their metal oxides reducing capability, charred wood enhanced ancient construction efforts in the flooded and swamp areas by bringing an environmental stability and water-damage resistance to building supports. About 1500 B.C., Charred wood was also applied for adsorption of odor from putrefying wounds. Newer sources indicated that about 450 B.C., charred wooden barrels were used to store drinking water, delaying the growth of bacteria and algae. A more modern usage of carbons started about 1700 A.D. when doctors began to see the advantage of chars in controlling bilious problems and odor from gangrenous ulcers. Those dates should be considered as rather approximate since it is rather difficult to find direct sources supporting the mentioned-above applications [1]. Nevertheless, the early 1900s was the time marking a major breakthrough in the application and further development of carbon-based materials [2,3,4,5].

A significant step forward was made by an inventor and chemist of Polish nationality, Rafal Ostrejko [2]. Various sources indicate his name also as Raphael von Ostreyko in German or Rapalas Osteika in Lithuanian. His inventions address the activation of charcoal by various agents to create porosity [2,3,4,5]. One of the first factories adopting the Ostrjeko’s activation processes was built in Ratibor in the German Empire (now Raciborz, Poland).

It was never a better time for the practical application of Ostrejko’s inventions. In 1914, World War I (WWI) started. It took 37 million casualties, and its effects would have been even more devastating to humanity if the inventions of Ostrejko would have not been put to work to save human lives. One of saddest realities of that war was the usage of Chemical Warfare Agents (CWAs). In the battlefields of WWI over 50 CWA were deployed leading to numerous deaths [6]. To protect against CWAs, gas masks were developed, and all fighting sides were involved in those efforts. Starting from 1915, German scientists found that activated charcoal used in filters provided a significant protection against many CWAs. The British started to use charcoal in 1916 (SBR mask), and Americans in 1917 used activated carbon from coconut shells in their CE type masks protecting against phosgene, tear gases and chlorine [6].

In the 20th century, owing to the invention of Ostrejko, activated carbons have gained more and more popularity mainly owing to their high surface area and porosity promoting physical adsorption. Depending on their physical form (granular, powders, or pellets), they have been widely applied in water/wastewater treatment, air purification, food and sweetener industry, mercury removal from flue gases, respirators, cigarette filters, water filters, automotive filters, in the pharmaceutical industry or in the chemical industry as catalysts or catalyst supports. In the majority of these applications, they were used either as adsorbents or separation media. They also saved numerous lives when applied orally as human body detoxifies or blood purifiers in dialysis equipment [7]. 

The last years of the 20th century and the beginning of 21st changed dramatically the view, applications and even names of activated carbons. In 1996, the Nobel Prize in Chemistry was awarded jointly to Robert F. Curl Jr., Sir Harold W. Kroto and Richard E. Smalley for the discovery of fullerenes, and in 2010, Andre Geim and Konstantin Novoselov were awarded the Nobel Prize in Physics for the discovery of graphene. These discoveries, and new applications based on them totally changed the perspectives and perception of carbon-based materials. All of these also happened in parallel with a developing research and application stress on nanosize, nanomaterials and related to this, nanotechnology. In this “Nanoworld”, a fraction of nanometer size of nanotubes or graphene layers in conjunction with their superior electrical conductivity or strength were very attractive features bringing paradigms of new unexplored fields where carbon-based materials could find their way to revolutionize science and engineering. Moreover, this, in conjunction with an involvement of physicists in carbon science besides that of chemists and chemical engineers, opened and broadened the applications of activated carbons in supercapacitors [8,9,10], lithium-ion batteries [11,12,13], advanced catalysts for energy production [14,15,16,17,18], sensors [19,20,21,22], photosensitizers [23], reactive adsorbents [24,25], and in many other high-tech applications. Related to those developments was a significant alteration in a nomenclature; activated carbon or microporous carbon started to be referred to as nanoporous carbon owing to the abundance of pores with sizes in the fraction of nanometers. Important for carbon science was also the progress in the development of research instrumentation, especially of high-resolution microscopes. With their help, scientists were able to see that in fact the walls of these nanopores are built of distorted graphene layers [26]. Thus, activated carbon aka “nanoporous carbons” could be considered as the “poor-person’s graphene”. The reason for the new name was probably not entirely related to the attractiveness of the “nano” word but also to the fact that many modern forms of carbons have been obtained without any traditional activation process.

Pores are a very unique feature and asset of nanoporous carbons. Even though they cannot be controlled to the same extent as those in nanotubes, the need for carbons with well-defined and abundant pores was a driving force for the development of methods of synthesis of ordered porous carbon (with pores mainly in the range of mesopores) [27,28,29] or 3-D hybrid carbon structures built from nanoforms of carbons [16]. The development of pores was also important for the advanced applications of carbon fibers or textiles, and some of them can reach high surface areas and pore volumes comparable to those in nanoporous carbons [30]. The pores in carbons, besides providing an extensive surface area and strong adsorption potentials, also affect the population of defects in graphene layers, and more porous carbons are expected to be more defective and to have a high number of exposed graphene edges, active in catalysis [31].

The importance of pores has been a driving force for the extensive search for the methods being able to quantify not only pore volumes [32] but also their sizes. Among them, a marked attention gained the Horvath –Kawazoe (HK) approach [33]. which used an average potential function inside the pores. This method was shown as inferior to modern Density Functional Theory Approaches [34,35,36,37], and here, significant contributions by Gubbins and coworkers [35], Neimark and coworkers [36] and Jagiello and coworkers [37] should be mentioned. In search for the method reflecting best the sizes of pores, various approaches have been explored including quench solid DFT [36] or NLDFT accounting not only for the heterogeneity of pore sizes but also for that of pore walls [38]. To get the most complete view of carbon porosity, various probe gases have been applied including N_2,_ Ar, CO_2_ [39] and quite recently O_2_ [40] or H_2_ [41]

In the analysis of the features of nanoporous carbons and their applications, their surface chemistry should not be neglected [42]. The reality is that in these complex materials, one cannot clearly distinguish specific effects of both these features on a target performance. Moreover, chemistry can be quite complex involving electron-donor and electron acceptor groups consisting of, in the majority of cases, oxygen, nitrogen, sulfur or phosphorus either incorporated to carbons rings or in sp^3^ configurations. While in the traditional applications of nanoporous carbons, nonspecific interactions were considered as most important and the extent of the carbon surface was a kind of “safety margin” granting their good or sufficient performance as adsorbents, the situation changed with the applications of carbons in *beyond adsorption* fields, where the electronic, optical or thermal properties started to play a dominant role.

In those *beyond adsorption* applications of nanoporous carbons, the effects of guest molecule-host adsorption (specific or nonspecific) interactions are often simplified and, in many cases, not accounted for. However, these pores and these interactions can significantly affect the performance. In spite of adding much more complexity to the explanation of the phenomena observed, they might govern the performance in a *silent* way, and pointing out this *silent* aspect is the objective of this minireview. Inspired by the application of graphene, we have focused our recent research efforts on studying the application of nanoporous carbons *beyond adsorption*. Namely, we have tested them as sensors [19,20,21,22,43,44] carbon dioxide (CO_2_ERR) reduction electrocatalysts [45,46,47,48] and oxygen reduction (ORR) electrocatalysts [49,50,51,52,53,54,55,56,57]. In all these applications, we have found indications that porosity and adsorption affect a target performance, and these effects could not be noticed on 2-D graphene-based materials owing to their lack of porosity. In all these applications, surface chemistry plays an important role adding to the complexity of a search for hypothesis supports. Nevertheless, even though one might argue that the sufficient proves on the effect of pores have not been provided, some indications of these effects certainly were found and described. Therefore, our intention is to rather point out the existence of these effects than to explain them in detail. The latter is a challenge, but that challenge might be worth taking up to advance nanoporous carbons’ modern applications and thus the development of new technologies. We have limited the scope of this minireview to the presentation and short analysis of the hypotheses and results tested in our laboratory, and only to the materials referred to as nanoporous carbons. Certainly, pores in other specifically designed novel carbons might also affect various processes, but addressing this is beyond the scope of this paper. We also do not focus on theoretical calculations, which, when applied, might deepen the understanding of the phenomena addressed here. 

## 2. Gas Sensing Enhancement by Adsorption the Pore System

During the last decade, graphene and nanotube have been widely tested as gas sensors [58,59]. Their significant asset for these applications is a high electrical conductivity, which changes in the linear way upon gas- graphene/nanotube interactions. Nevertheless, soon it was found that high sensitivity was not a sufficient feature, when selectivity was missing. That deficiency of pristine and defect-free forms of carbons directed scientists to the surface modifications of these materials, which involved an incorporation of heteroatoms to the carbon’s matrix and addition of polymers or metal oxides [60,61,62,63,64,65]. Specific interactions of these additives with target molecules provided selectivity, even though sensitivity could be compromised to some extent. The efforts taken were often time and resources consuming and led basically to defective nanoforms of carbons or carbon composites. This directed our attention to nanoporous carbons [18,19,20,21,22,43,44]. The motivation behind this was that (1) they are built of defective graphene layers; (2) they have some electrical conductivity; (3) they are full of defects; (4) oxygen naturally exists on their surface, and other heteroatoms such as nitrogen can be easily introduced to their matrix in various configurations [42]; (5) they are much less expensive than graphene and carbon nanotubes are; and last but not least, (6) they have pores which, when filled with target gas, can change the conductivity of the sensing films. The suitability of carbons to detect gases, especially toxic ones as H_2_S and NH_3_ was also based on our extensive experience in evaluation of nanoporous carbons as reactive adsorbent for these gases [66,67].

**Figure 1:**
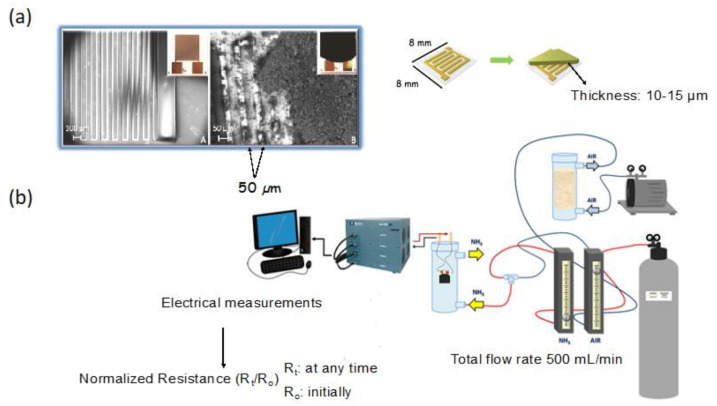
(**a**) A chip without and with a carbon film (upper panel) and (**b**) experimental set up to test the sensing response. Adapted with permission form Reference [20]. Copyright 2016, The Royal Society of Chemistry.

The testing system consisted of sensing chips covered with a thin film of carbon, placed in the chamber purged with target gas of various concentrations (Figure 1). Upon exposure to target gas, the conductivity of the chips was measured, and a relative change in this parameter was analyzed. Preparing the conductive film of carbon of a high structural integrity was a time-consuming challenge.

As the first challenge, gas ammonia was chosen, and the sensing abilities of polymer derived carbons were tested, and differences observed in the response were linked to the differences in ammonia interactions with the surface and its adsorption [19]. Then, extensive studies were done on wood-based carbon, BAX-1500 (Ingevity) [20]. The carbon was chosen based on its relatively high capacity to adsorb ammonia [56]. The carbon was highly porous (micro/meso), and our previous studies indicated that its relatively high surface acidity and abundance of oxygen groups on the surface attracted ammonia in a relatively high quantity [67]. To even further increase the ability of this carbon to interact with ammonia, it was mildly oxidized with HNO_3_. Knowing that ammonia chemically reacts with some groups of this carbon and that for a reversible sensing physical adsorption should rather be the target, the surface of both carbons, BAX and BAX-O, was initially stabilized by the exposure of the chips to ammonia until the resistance reached a constant level. Interestingly, for BAX, the normalized resistance upon exposure to NH_3_ increased and for BAX-O-decreased. Air purging upon exposure resulted in opposite effects and decreased 13% the normalized resistance of BAX and increased 20% that of BAX-O. That process removed physically adsorbed ammonia, and the differences in its extent were linked to the differences in porosity. As seen from Table 1, even though the oxidation of BAX decreased the overall volume of pores, it markedly increased the volume of ultramicropores (V_<0_._7 nm_), and these pores should be the most active in physical adsorption of ammonia at ambient and dynamic conditions. After exposure to ammonia and high vacuum outgassing, the minimal changes in porosity for both carbons were recorded, suggesting that the before-porosity-measurement pretreatment (heating at 120 °C and high vacuum) removed the majority of ammonia either chemisorbed and/or physisorbed on the surface.

The detailed analysis of the surface chemistry by XPS indicated that the content of oxygen on the surface of BAX increased twice after oxidation (from ~9% to ~18%). Interestingly, ~2.6 at. % of N was introduced to the surface as a result of the nitric acid treatment. The majority of new oxygen species was in O-C bonds (in BAX-O=C predominated), and nitrogen was mainly in Ph-NO_2_ species. After exposure to ammonia, NH_4_^+^ ions were detected, and the surface of BAX was more reactive towards the formation of these compounds. These trends were consistent with the expected surface reactivity based on the chemical environments of both surfaces. They were also reflected in the extent of the changes in the normalized resistivity of the chips upon stabilization and air purging discussed above.

The opposite trends in both chips’ responses upon exposure to ammonia were a puzzle. To explain this, carbons were treated as semiconductors and the Mott–Schottky plots were constructed from the results of impedance spectroscopy [68,69]. Even though both charge carriers (holes and electrons) were present in both samples, the slopes indicted that BAX was more a p-type material and BAX- more an n-type. While carbons are usually p-type materials [58], oxidation with nitic acid and the incorporation NO_2_ moieties increased the population of electrons. Thus, upon exposure to ammonia, which is an electron donating species, the resistivity of BAX increased (electrons interacted with holes decreasing their population) and that of BAX-O decreased (population of charge carrier increased). Nevertheless, we have recognized that for the performance of the sensors, the relative change in the resistivity upon exposure to the target gas and the reproducibility of the signal are important and not the direction of that change.

**Figure 2:**
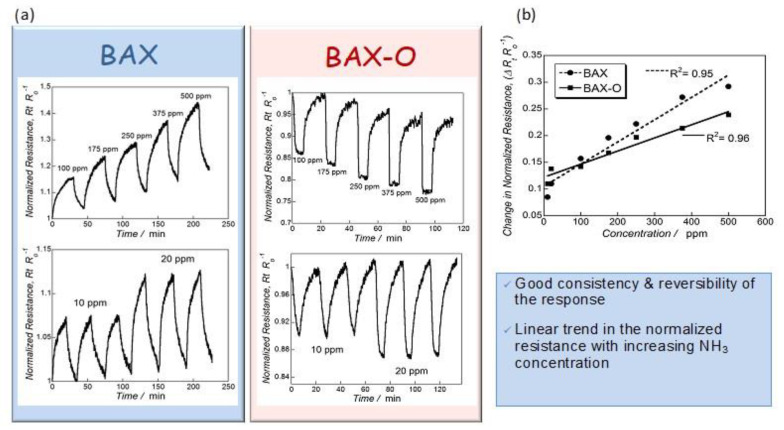
(**a**) Dependence of BAX and BAX-O responses (upper panel) on the NH_3_ concentration and reproducibility of the resistance changes (lower panel) and (**b**) the linear response of the sensors on NH_3_ concentration (upper panel). Adapted with permission from Reference [20]. Copyright 2015, The Royal Society of Chemistry.

**Figure 3:**
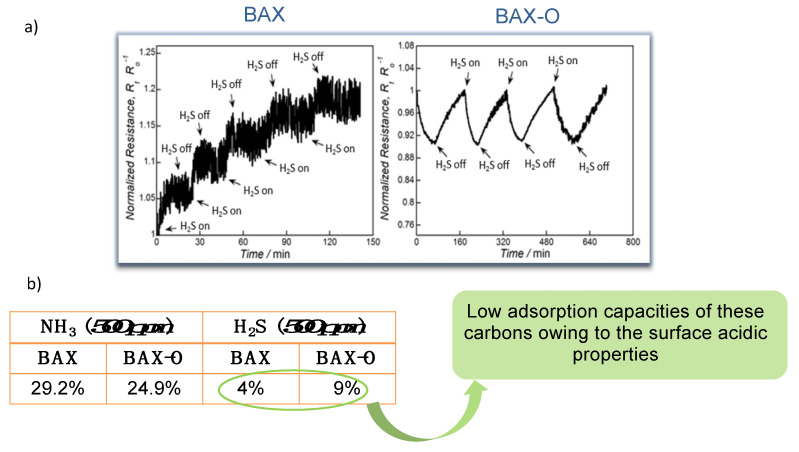
(**a**) Response of sensors to H_2_S and (**b**) normalized resistance changes upon exposure to 500 ppm of NH_3_ and H_2_S (in %). Adapted with permission from Reference [20]. Copyright 2015. The Royal Society of Chemistry.

Figure 2 shows the actual response of the chips to ammonia. Up to 30% change in the response was recorded, and this value was similar to that found on modified graphene [59,60,61,62,63,64]. Even though these results showed the sufficient sensitivity of the nanoporous carbon chips, still the selectivity was not established. For this purpose, we tested the response of the chips to hydrogen sulfide. It is also a small molecule gas (~0.3 nm) and electron donor, as ammonia is, but of an acid nature. The response of the chips was very small (Figure 3) and linked to limited physical adsorption of H_2_S on the surface owing to its acidic character [66]. Even though in some cases, especially for nitrogen modified nanoporous carbons, some enhanced sensitivity to H_2_S was measured (~12% change in the normalized resistivity) [22], and it was comparable to that of ammonia; the response time was 20 times longer (42 min vs. 2 min). Such as a response difference was a general trend, and it was certainly affected by an adsorption equilibrium in the carbon pores and specific interactions with the functional groups/reactive adsorption

Since the sensors are expected to work in ambient conditions where presence of humidity can affect their response, our chips were also tested in a moist environment [21]. It was found that while on hydrophobic carbons water did not affect the performance, the sensitivity increased over 100% when chips made of hydrophilic carbons were used. This effect was linked to the adsorption of water in the pore system and its reactivity with ammonia and formation or ions contributing to the electrical conductivity.

In our research on porous carbon as gas sensors, the effects of diverse surface properties were tested [19,22,43,44,70]. We have found that both porosity and surface chemistry are important. Adsorption and the presence of the target gases or vapors in the pore system certainly affected the conductivity of the chips [71]. In sensing of gases of distinctive chemical features, the effect of specific interactions was of paramount importance. Nevertheless, the physical interactions and retention of gases with strong adsorption forces affected the conductivity of the film by contributing to the overall effect of an adsorbed condense phase electronic properties and also by specific electronic interactions with the pore walls. Various research reports have been recently published stressing the importance of the pore system in carbon-based materials for sensing properties [72,73,74].

## 3. Ultramicropores as Nanoreactors for CH_4_ Formation during CO_2_ Electroreduction

In search for alternative sources of energy and for the ways to mitigate/decrease global warming, CO_2_ electroreduction occupies one of the first strategic research directions. Even though transitions metals, especially those based on copper, were found to be efficient and selective electrocatalysts for this process [75,76], the search to replace metals continues with the emphasis on carbon materials. Chemically modified graphene and nanotube gained considerable attention [77,78,79]. Especially those with nitrogen species such a pyridines and quaternary nitrogen incorporated to the carbon matrix [68]. These sites were found as catalytically enhancing CO_2_ reduction on a cathode. This process is complex and requires a multielectron transfer and large overpotential. Moreover, the CO_2_ reduction potential overlaps with that of hydrogen generation in an aqueous electrolyte. Various products of the reduction have been reported as formed on nanoforms of carbons, including CO, various hydrocarbons, formate, formic acid and various alcohols [80,81,82].

Based on the published findings on the importance of nanocarbon specific surface features for CO_2_ electroreduction [75,76,77,78,79,80,81,82], we decided to check the effect of porosity on the performance of polymer derived carbons, which contained S (CPS; 1 at % of S) and S and N (CPSN; ~3.5 at % of both S and N) heteroatoms [48]. While sulfur was mainly in thiophenic configurations, pyridines were in majority in the N-modified sample. The carbons were ash-free so the effect of the trace of metals could be excluded from the interpretation of the results. For comparison, the BAX sample, mentioned above, have been also tested. The parameters of pore structure are present in Table 2.

**Figure 4:**
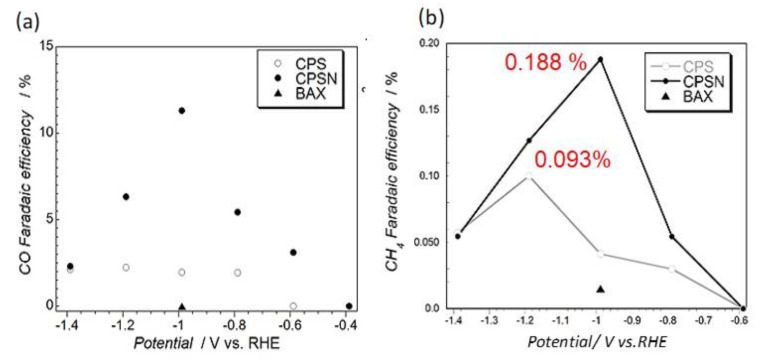
(**a**) Faradaic efficiency for CO and (**b**) for CH_4_ formation on the carbons tested. Adapted with permission from Reference [48]. Copyright 2016, Wiley.

Even though the Faradaic efficiency for hydrogen evolution was very high at the potentials tested, CO and CH_4_ were also detected. The measured efficiencies for the CO_2_ reduction products on our carbons are presented in Figure 4. The most intriguing result of these experiments was the detection of CH_4_. Its formation was not reported on graphene or carbon nanotubes of modified surface chemistry. Its formation in the electrochemical process is not very straightforward since it requires the transfer of eight electrons. This directed us to take a closer look at the effects of porosity. Certainly, small pores, especially those close in size to CO_2_ molecule, should strongly attract both CO_2_ and formed CO. Besides, H_2_ was also generated and its adsorption was expected. Knowing that in confined nanospace with adsorbed molecules very high pressure can be generated [83], directed us to look at methane formation as a pseudo-Fischer–Tropsch process [84]. It is plausible to assume that strong adsorption of CO and high pressure result in C=O bond splitting, which was followed by the acceptance of hydrogen, which was also adsorbed in pores [85] leading to CH_4_ formation. 

**Figure 5:**
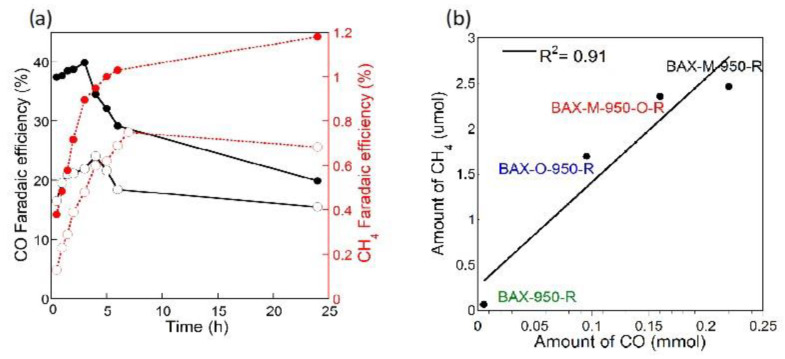
(**a**) Increase in the Faradic efficiency for CO and CH_4_ formation on nitrogen-modified commercial carbon upon duration of a catalysts electroreduction process prior to CO_2_ERR, and (**b**) dependence of the amount of CH_4_ formed upon the amount of CO formed. Adapted with permission from Reference [47]. Copyright 2017, Elsevier.

**Figure 6:**
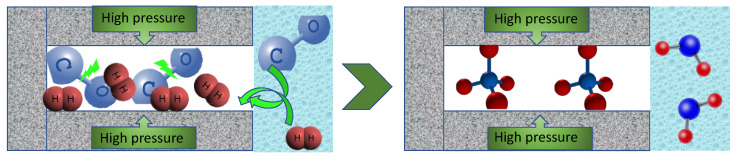
Pore-mediated process of CH_4_ formation from CO and H_2_.Steps: (1) Both CO and H_2_ are formed at the potential of CO_2_ reduction; (2) they are withdrawn from an aqueous phase and adsorbed in small pores similar in sizes to their molecules due to the strong adsorption potential; (3) in these pores, pseudo Fischer–Tropsch process takes place owing to high pressure and the proximity of the molecules (splitting of bonds and hydrogenation); (4) methane stays adsorbed in pores and formed water molecules are attracted to the aqueous phase in larger pores.

To further study CO_2_ electoreduction on nanoporous carbons, we used a variety of commercial and lab synthesized structurally and chemically heterogeneous catalysts [45,46]. Generally, a modification with nitrogen and surface electroreduction increased the Faradaic efficiency for both carbon monoxide and methane formation (Figure 5, left panel). CH_4_ was formed on all porous catalysts although a direct relationship between the amount of CH_4_ formed and porosity (volume or sizes) was not found. On the other hand, the results indicated the linear relationship between the amount CH_4_ formed and that of CO suggesting that the formation of CO is an intermediate step for that of CH_4_, which indeed follows our hypothesis on the pore-mediated pseudo-Fischer–Tropsch catalytic process. That apparent lack of relationship between the porosity and the amount of CH_4_ formed might be caused by the important influence of heteroatom-based catalytic sites on the CO formation. Thus, the efficiency of CH_4_ formation is dependent, although indirectly, on the activity of the catalytic sites on the carbon surface. The visualization of the pseudocatalytic effects of pores on CO_2_ electroreduction and formation of methane is presented in Figure 6. 

The results of CO_2_ electroreduction described above stress the role of pores and adsorption potential in this process. Due to this contribution, the pores provide an entirely different than electrochemistry component to the mechanism, and thus, their influence should not be neglected. That effect of porosity has been also indicated recently in the studies of various CO_2_ carbon-based electrocatalysts [86,87,88]. Taking it into account might help to develop even more efficient CO_2_ reduction processes. 

## 4. Ultramicropores as Pseudocatalytic Centers Enhancing Oxygen Electroreduction 

**Figure 7:**
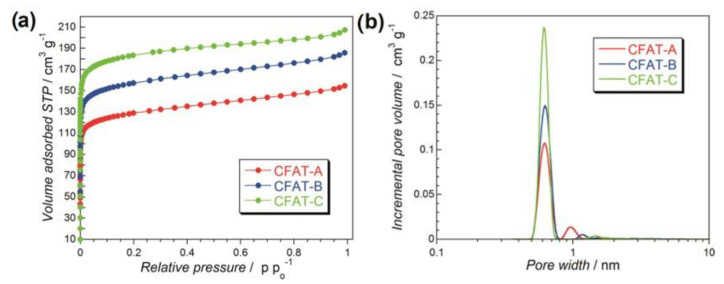
(**a**) Nitrogen adsorption isotherms and (**b**) pore size distributions for polyHIPE-derived carbons. Reprinted with permission from Ref. [53], Copyright 2016, American Chemical Society.

Following the applications of modified graphene for ORR and recent findings on the importance of these materials’ surface features for this process [89,90,91], we have started to apply nanoporous carbons as oxygen reduction electrocatalysts [49,50,51,52,53,54,55,56,57]. The experiments were performed in an alkaline electrolyte. To clearly underline the effects of porosity, three hydrophobic carbons obtained from polyHIPE (polymerized high internal phase emulsion) and containing micropores of the same sizes but differed in their volumes were chosen [53]. They were referred to as CFAT-A, -B, and -C, and the volume of ultramicropores increased from A to C, as seen in Figure 7. All samples, in spite of ~9 at % of oxygen were found hydrophobic with ethers as predominant surface groups.

**Figure 8:**
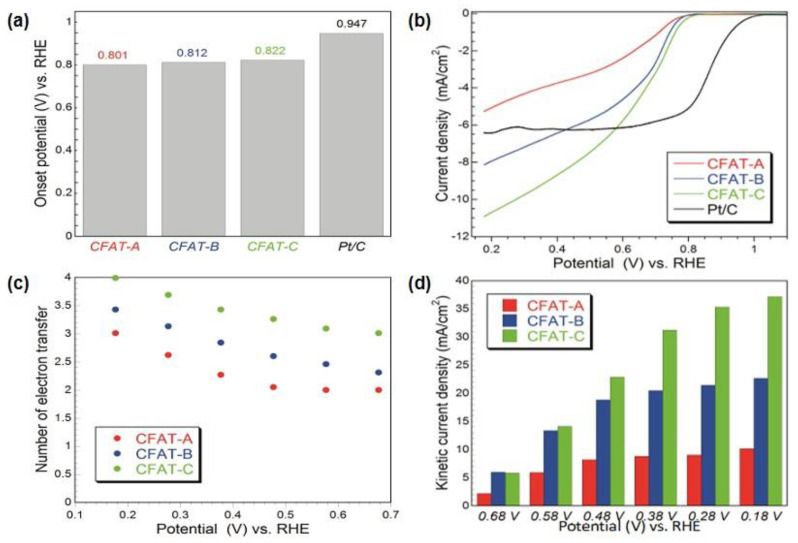
For the materials studied: (**a**) onset potentials; (**b**) linear sweep voltammograms on the modified glassy-carbon RDE in O_2_-saturated 0.10 MKOH at 2000 rpm and scan rate of 5 mV/s; (**c**) number of electron transfers versus potential; (**d**) kinetic current density. Reprinted with permission from Reference [53], Copyright 2016, American Chemical Society.

The performance of the polyHIPE derived carbons as ORR electrocatalyst is presented in Figure 8. Even though the onset potential measured was less positive than that on Pt/C (Figure 8b), the current density was higher, and that current density along with the onset potential, number of electron transfer and kinetic current density increased with an increase in the volume of ultramicropores. This directed us to hypothesize that a hydrophobic surface (ethers are hydrophobic [92]) and small pores advance the oxygen reduction reaction by providing sites for strong adsorption of oxygen upon its withdrawal from an electrolyte, and in these pores, the reduction process is enhanced.

**Figure 9:**
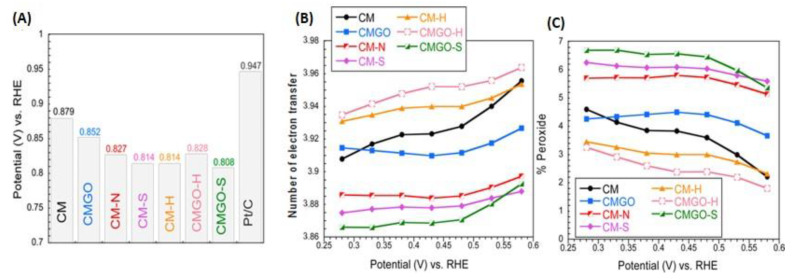
Comparison of the (**A**) onset potential, (**B**) the number of electron transfer, and (**C**) the percentage of oxygen reduced to peroxide. Reprinted with permission from Reference [54]. Copyright 2017, American Chemical Society.

To further support our hypothesis, we expanded the research to porous carbons containing heteroatom—based groups known to catalytically enhance ORR [54,55,56,57]. The introduction of N, S, and B to the carbon matrix was our goal, and the spatial geometry of those groups and the modification means used rather excluded their existence in ultramicropores with size less than 0.7 nm. Thus, the latter were expected to be predominantly built of carbon and be of a hydrophobic nature. A carbon foam (CM) with a heterogenous pore structure was chosen, which, besides micro- and mesopores, contained also macropores [54]. Its porosity was modified by the deposition of GO in its pores, and its chemistry- by a heat treatment (H) or by a chemical/thermal introduction of N and S groups to the surface. The size of ultramicropores remained mainly unaffected, although their volume decreased upon the treatment. The range of the oxygen content was from 8–19%, that of N- 3.8 at % and S-1%. The volume of ultramicropores decreased markedly (up to 50% from 0. 251 to 0.123 cm^3^/g) upon the introduction of sulfur and/or nitrogen groups which were known as advancing the ORR process. Interestingly, the best preforming samples were those without heteroatoms and having the high volume of ultramicropores and hydrophobic surface (Figure 9). This provided another support for our hypothesis on the enhancing effect of small pores for ORR.

That effect of nitrogen modification of ordered mesoporous carbons of a marked volume of ultramicropores on the efficiency of ORR has also been studied [56]. Nitrogen in this case was introduced by a thermal urea treatment (from 800–950 °C). The carbons tested showed the good performance with the number of electron transfer 3.7–3.9. When the effect of porosity was analyzed in detail in this process, both the volumes and the predominant sizes of ultramicropores were taken into consideration to account for extensive and intensive effects (based on their influence of physical adsorption of oxygen), the dependence of the number of electron transfer on the volume of ultramicropores showed an increasing trend for the nitrogen-modified N-modified) carbons (R^2^ = 0.99) and a decreasing one for the non-N-modified samples (R^2^ = 0.87). The predominant size of ultramicropores strongly affected the number of electron transfer for N-modified carbons (R^2^ = 0.99) and smaller size led to the larger number of electron transfer, n. On the other hand, no clear effect of the size was found for the non-nitrogen modified samples. These results suggested that even though the pore size and volume affect the efficiency of ORR, the effects are different for different groups of carbons, which might be related to different mechanisms involved in O_2_ reduction. The difference in the trend of the dependence of the kinetic current on surface area for N-modified and non-N-modified carbons were also found by Pereira and coworkers [93].

**Figure 10:**
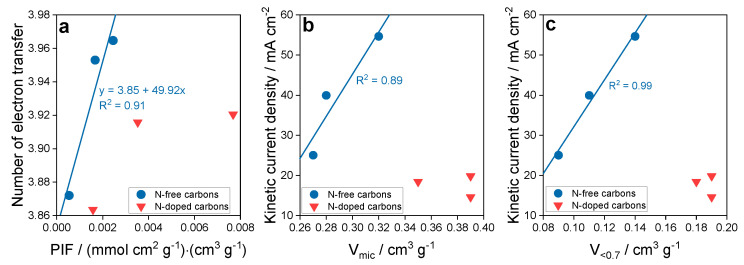
(**a**) Dependence of the number of electron transfer on PIF, and dependence of the kinetic current density on (**b**) volume of micropores and (**c**) volume of ultramicropores. Reprinted with permission from Reference [57], Copyright 2019, The Royal Society of Chemistry.

The effect of ultramicropores on ORR was also studied on another group of synthetic carbons obtained from sucrose and KIT-6 silica as a template [57]. In that case, ammonia was a nitrogen source and the samples were heated between 600 and 950 °C. Non-nitrogen modified samples were the initial one, oxidized and reduced by heat treatment at 950 °C. While the treatment with ammonia practically did not affect the porosity, the nitrogen-free samples showed some differences in this quantity (Figure 10). These differences and similarities were in fact reflected in the parameters describing the efficiency of the ORR process. Both, the number of electron transfer and onset potential were more favorable on the non-N-modified samples. Knowing that (1) ultramicropores, even though very important, do not exist in separation from other features of the carbon surface, and that (2) their hydrophobicity would be totally useless to attract and adsorb oxygen when an aqueous electrolyte with dissolved oxygen would not be able to reach them, we assumed an expanded level of the complexity of the interactions/process. To account for all of these, we proposed a Pore Influence Factor (PIF) as a product of the volume of ultramicropores and the ratio of the number dissociating groups to ECSA (electrochemically active surface area). While ECSA reflects the accessible surface to an electrolyte and the dissociating group—a hydrophilicity level in larger pores enhancing the transport of the electrolyte through the pore system, V_<0_._7 nm_ promotes an oxygen withdrawal and adsorption and then reduction. The analysis of the dependence of the number of electron transfer on PIF is presented in Figure 10 where a linear tend was found for the non-N-modified samples. For those treated with ammonia, no relationship was uncovered since the samples were very similar in all aspects and also behaved similarly in ORR. The linear dependence of the kinetic current density of the non-N-modified samples of V_mic_ and V_<0_._7_ (volume of micropores and pores smaller than 0.7 nm, respectively) was also found with the stronger affect for the latter pore sizes. This further stresses the importance of the oxygen adsorption forces in these pores.

Interesting results further supporting our hypothesis were also found when the highly porous carbons modified with nitrogen and boron were tested as ORR [55]. The samples markedly differed in their surface areas (390 to 1560 m^2^/g) and pore volumes (V _<0_._7 nm_: 0.01–0.15 cm^3^/g) and in the content of heteroatoms (N: 0.8–11 at. %; S:0.4–0.5 at. %; B:0–10.9 at. %) and, subsequently, in the catalytic centers known to advance ORR. Although all samples performed well and n was almost 4 and the onset potentials similar to that on Pt/C, the best performing samples were those of the highest porosity, and the sample with practically no heteroatoms (only traces) but with the highest surface area outperformed those with the high populations of the catalytic centers. We concluded that even though the chemical/thermal treatment introduced the catalytic centers, owing to a marked decrease in the surface area, these centers, even though very active, were not able to compensate the effect of the enhanced porosity on ORR.

**Figure 11:**
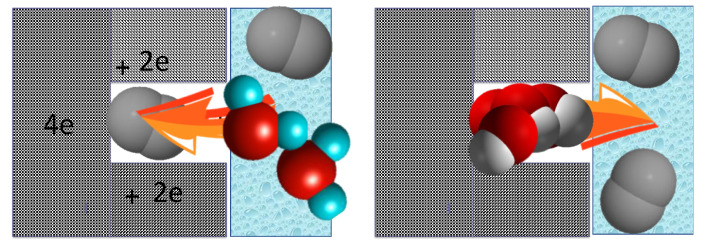
Schematic view of the involvement of ultramicropores/O_2_ adsorption in ORR. Reprinted with permission from Reference [57]. Copyright 2019, The Royal Society of Chemistry.

The visualization of the proposed ultramicropore-mediated mechanism of ORR on nanoporous carbons is presented in Figure 11. It shows that when an electrolyte with dissolved oxygen reaches the entrance of ultramicropores, oxygen is attracted there and owing to the strong adsorption potential and the influence of the cathodic current O=O bonds splits, 4 electrons are accepted, and protonation takes place owing to the proximity of the water phase. Formed OH^−^ ions do not linger in the pores, and they are rather attracted to the aqueous phase leaving the sites/ultramicropores for further advancing the ORR process on these materials. Those effects of small pores on ORR were also recently indicated in other published works including those by Pereira et al. [93], Cazorla-Amoros et al. [94], Freire et al. [95] and Luo et al. [96].

## 5. Conclusions

In conclusion, even though nanoporous carbons are very complex materials, they provide the diversity and heterogeneity of surface features which can prove to be very important for the applications reaching *beyond adsorption*. A few examples discussed here include sensing, carbon dioxide electroreduction and oxygen reduction reaction. All three are cutting-edge applications of carbons owing to their electrical conductivity and abundance of defects beneficial for catalytic processes. In these fields, nanoporous carbons, especially those with small pores, are often considered as not very suitable materials owing to the mass transfer limitations. It is certainly true when traditional catalysis is considered and the sites in small pores are restricted to the reacting molecules. However, in our approach presented in this minireview, we have shown *silent* benefits of small pores in terms of them providing adsorption forces, which, in turn, might advance other main processes taking place on the surface. We believe that the processes in which nanoporous carbon are applied, and not necessary those focused on adsorption but those fully utilizing of the carbons’ nanopore potential, can be markedly advanced by comprehending the confined space/pore effect on the final performances. However, that comprehension is a challenge in the case of nanoporous carbons, owing to the structural and chemical complexity mentioned above and a strong synergy and interconnections of the surface effects. Achieving that understanding is certainly more challenging than addressing the behavior of relatively simple graphene, but in our opinion, it is worth exploring. 

## Figures and Tables

**Table 1:** Parameters of the pore structure of BAX and BAX-O and their counterparts exposed to ammonia in dynamic conditions. S_NLDFT_-surface area calculated using a NLDFT approach [38]; V_<0_._7 nm_-volume of ultramicropores; V_mic_-volume of micropores; V_meso_-volume of mesopores, V_t_-total pore volume, calculated from nitrogen adsorption isotherms). Adapted with permission from Reference [20]. Copyright 2015, The Royal Society of Chemistry.

Sample	S_NLDFT_ [m^2^/g]	V_<0_._7 nm_ [cm^3^/g]	V_mic_ [cm^3^/g]	V_meso_ [cm^3^/g]	V_t_ [cm^3^/g]
BAX	1549	0.086	0.617	0.756	1.373
BAX-O	1408	0.147	0.551	0.532	1.082
BAX-E	1616	0.090	0.648	0.793	1.441
BAX-O-E	1427	0.137	0.556	0.532	1.088

**Table 2:** The parameters of pores structure calculated from nitrogen adsorption isotherms. Adapted with permission from Reference [48]. Copyright 2016, Wiley.

Sample	S_BET_ [m^2^/g]	V_t_ [cm^3^/g]	V_meso_ [cm^3^/g]	V_<0_._7 nm_ [cm^3^/g]	V_<1 nm_ [cm^3^/g]	V_mic_ [cm^3^/g]
CPS	1523	1.354	0.728	0.235	0.398	0.626
CPSN	1332	1.307	0.740	0.271	0.394	0.567
BAX	1541	1.074	0.554	0.159	0.277	0.520
